# Laboratory assays reveal diverse phenotypes among microfilariae of *Dirofilaria immitis* isolates with known macrocyclic lactone susceptibility status

**DOI:** 10.1371/journal.pone.0237150

**Published:** 2020-08-06

**Authors:** Jeba R. J. Jesudoss Chelladurai, Katy A. Martin, Krystal Chinchilla-Vargas, Pablo D. Jimenez Castro, Ray M. Kaplan, Matthew T. Brewer

**Affiliations:** 1 Department of Veterinary Pathology, Iowa State University College of Veterinary Medicine, Ames, IA, United States of America; 2 Department of Infectious Diseases, College of Veterinary Medicine, University of Georgia, Athens, GA, United States of America; 3 Grupo de Parasitologia Veterinaria, Universidad Nacional de Colombia, Bogotá, Colombia; University of California Riverside, UNITED STATES

## Abstract

Prevention of canine heartworm disease caused by *Dirofilaria immitis* relies on chemoprophylaxis with macrocyclic lactone anthelmintics. Alarmingly, there are increased reports of *D*. *immitis* isolates with resistance to macrocyclic lactones and the ability to break through prophylaxis. Yet, there is not a well-established laboratory assay that can utilize biochemical phenotypes of microfilariae to predict drug resistance status. In this study we evaluated laboratory assays measuring cell permeability, metabolism, and P-glycoprotein-mediated efflux. Our assays revealed that trypan blue, propidium iodide staining, and resazurin metabolism could detect differences among *D*. *immitis* isolates but none of these approaches could accurately predict drug susceptibility status for all resistant isolates tested. P-glycoprotein assays suggested that the repertoire of P-gp expression is likely to vary among isolates, and investigation of pharmacological differences among different P-gp genes is warranted. Further research is needed to investigate and optimize laboratory assays for *D*. *immitis* microfilariae, and caution should be applied when adapting cell death assays to drug screening studies for nematode parasites.

## Introduction

*Dirofilaria immitis* is the causative agent of canine heartworm disease, which is capable of causing debilitating cardiovascular and respiratory signs in domestic dogs, cats, wild canids, and mustelids including ferrets. Control and prevention of *D*. *immitis* largely relies on macrocyclic lactone (ML) chemoprophylaxis. This strategy prevents the development of adults from 3^rd^ stage larvae of *D*. *immitis* following infection by L3s from mosquito intermediate hosts. Remarkably, this approach has been largely successful for decades in the United States. Over the past ten years, numerous lack of efficacy (LOE) reports have been made by veterinary practitioners [1–3]. This is certainly an alarming development. Subsequently, ML resistance in *D*. *immitis* has been confirmed by establishing infections in dogs despite strict ML prophylaxis under controlled conditions [3].

The detection of bona fide ML resistance in *D*. *immitis* infected animals is a major clinical challenge. The only patient-side test that can predict drug resistance is the microfilaria suppression test which involves measuring the number of microfilariae in blood before and after ML treatment [4,5]. Laboratory-based larval migration inhibition assays measuring the ability of L3 to migrate through a fine mesh in the presence of drugs have also been described [6,7]. However, motility measurements are not capable of differentiating between resistant and susceptible strains [7,8]. Microsatellite-based genetic assays are being currently evaluated to identify resistant isolates of *D*. *immitis* from clinical samples, but are time-consuming and expensive [9]. However, no laboratory tests are available for determining drug susceptibility status of *D*. *immitis* obtained from clinical specimens such as canine blood. Thus, there is a need to develop laboratory assays that can be used to quickly and accurately detect ML resistance. Ideally, such an assay would utilize microfilariae from the peripheral blood of infected dogs and would be able to compare strain susceptibilities *in vitro*.

Laboratory assays that detect nematode mortality have been applied inconsistently among different clades of nematodes. For example, cessation of motility is often used to measure nematode death in basic science or drug screening studies [10]. Yet, there are many examples of parasites that continue to be motile despite being subjected to concentrations of drugs that are well above those reached in the tissues of a treated animal [8]. At the same time, our lab and others have noted that some nematodes lack obvious movement, but are still alive, often resuming motility following light or heat stimuli [11]. In addition, many drug discovery studies employ methods developed for detecting death in eukaryotic cell cultures without a clear acceptance of any particular method. Years of research have evaluated numerous drug susceptibility assays such as the larval motility inhibition test, the larval development test, the egg hatch assay and others [12–14]. Yet, there is not a single accepted laboratory assay for detection of resistance in nematodes. Both investigators seeking “death phenotypes” in drug development studies and clinicians facing treatment decisions would benefit from an improved method for detecting and quantifying drug resistance.

Spectrophotometric, colorimetric, densitometric and image-based assays have been used to evaluate helminth viability during *in vitro* drug studies [15,16]. Yet, from a clinical point of view, no standard method is available for quantification of drug resistance across nematode taxa. In contrast, minimum inhibitory concentrations are widely used to provide a standardized measure of antimicrobial resistance in bacteria [17]. For *D*. *immitis*, it is not clear what *in vitro* assays can be useful predictors of clinical drug susceptibility. Herein, we evaluate biochemical methods relying on membrane permeability, metabolic activity, and P-gp activity as surrogate phenotypic measures of ML susceptibility. These assays were selected as they could possibly be adapted to a wide range of clinical settings with access to a spectrophotometric plate reader. In this study, we hypothesized that these biochemical assays could differentiate between microfilariae from different *D*. *immitis* strains bearing a known drug susceptibility status. To test this hypothesis, we co-incubated microfilariae with different MLs and subjected them to common assays used to detect cell death.

## Materials and methods

### Ethics

The experiments performed for this study received research approval from Iowa State University protocol IBC-19-008.

### Parasites

Heparinized dog blood with live *D*. *immitis* microfilariae, strain Missouri (ML-susceptible, NR-48907) and strain JYD-27 (ML-resistant, NR-49172) were obtained from the NIH/NIAID Filariasis Research Reagent Resource Center. The Missouri isolate was originally isolated in 2000 and has been maintained without ML preventatives [18]. Heparinized blood from dogs experimentally infected with the ML-resistant strains Metairie and Yazoo were obtained from the University of Georgia [8].

All assays were carried out within 72 hours of receiving the blood samples. Microfilariae were isolated from the blood using a protocol modified from [11]. Briefly, 1 mL of blood was added to 10 mL of filter-sterilized 0.2% saponin in MilliQ water, incubated in a 37°C water bath for 15 min and centrifuged at 350 x g for 5 min at room temperature. The supernatant was removed and the microfilariae in the pellet were washed twice with 1x Dulbecco’s phosphate buffered saline. The pellet was resuspended in pre-warmed sterile 1x RPMI 1640 without antibiotics. Microfilariae in the suspension were enumerated in three 10 μL aliquots for each sample and used the same day.

### Assay design

Assays were performed in 96-well flat-bottomed plates (Corning). Microfilariae of each isolate were simultaneously tested on each plate whenever possible. Each test drug dilution had technical duplicates for each isolate and each well contained 300 total microfilariae with or without dilutions of MLs in a total volume of 200 μL. ML solutions were made by diluting drugs in 0.1% DMSO. Every plate had 3 technical replicates of three experimental controls: (1) 300 microfilariae killed in a heat block at 95°C for 1 min, (2) 300 live microfilariae with no drugs and (3) 300 microfilariae with drug vehicle (0.1% DMSO) only. To determine background values for each assay, each plate also had 6 wells containing only assay reagents with no parasites. For drug susceptibility experiments, each well contained 300 microfilariae co-incubated with dilutions of MLs in RPMI1640 at 37°C with 5% CO_2_ for 1 hr, followed by specific assays described below. Experiments were typically first conducted with Missouri and JYD-27 isolates due to availability and then investigated further with all four isolates. In the case of MTS and ATP assays, further investigation with all four isolates was not pursued.

### Trypan blue assay

To optimize the trypan blue assay, dilutions of the dye were incubated with different numbers of live microfilariae, incubated for 3 min at room temperature and absorbance measured at 590 nm with a spectrophotometer (M2 Spectramax). For drug susceptibility assays, 20 μL of 0.4% trypan blue solution in 1x Dulbecco’s PBS was added to each well and the plate was incubated for 3 min at room temperature followed by absorbance measurement in a spectrophotometer at 590 nm. Trypan blue assays were conducted four times with technical duplicates of test wells and technical triplicates of controls.

### Propidium iodide assay

Following ML incubation, 10 μL of 1 mg/mL aqueous solution of propidium iodide (Alfa Aesar) was added to each well and the plate was incubated at 37°C with 5% CO_2_ for 15 min. Fluorescence from each well was measured in a spectrophotometer at excitation wavelength of 535 nm and emission wavelength of 617 nm. The assay was conducted three times, with technical duplicates of test wells and technical triplicates of control wells.

### Resazurin- resorufin assay

As a measure of metabolism, resazurin reduction assays were performed whereby resazurin is metabolized to fluorescent resorufin. 20 μL of resazurin solution (CellTiterBlue, Promega) was added to each well and the plate was incubated at 37°C with 5% CO_2_ for 1 hr. Fluorescence of the reduced product resorufin was measured in a spectrophotometer at excitation wavelength of 579 nm and emission wavelength of 584 nm. The assay was conducted three times, with technical duplicates of test wells and technical triplicates of control wells.

### MTS tetrazolium assay

MTS assays were conducted with the colorimetric reagent—MTS tetrazolium in the presence of phenazine methosulfate (PMS). 20 μL of combined MTS/PMS solution (CellTiter96, Promega) was added to each well and the plate was incubated at 37°C with 5% CO_2_ for 1 hr. Absorbance of soluble formazan bio-reduced from MTS was measured in a spectrophotometer at 490 nm wavelength. The assay was conducted three times, with technical duplicates of test wells and technical triplicates of control wells.

### ATP luciferase assay

ATP production was measured using a commercial luciferase assay (CellTiterGlo, Promega). Following ML incubation, microfilariae were washed with 1x Dulbecco’s PBS, lysed with 0.5 mm beads (Zymo Bashing Beads) in a bead beater for 5 min, centrifuged for 5 min at 1600 x g, and 100 μL of lysate supernatant transferred to opaque walled 96 well plates. Incubation with lysis buffer in the kit failed to lyse microfilariae. 100 μL of the recombinant luciferase reagent was added to each well and the plate was incubated at room temperature for 30 min.”Glow type” luminescence resulting from the interaction of ATP with luciferase was measured in a spectrophotometer with 30 luminescent reads integrated over 5–10 min to obtain the final value. ATP was serially diluted to make a standard curve.

### Rhodamine 123 assay

ATP binding cassette B1 transporter (P-glycoprotein) activity was measured by incubating microfilariae with the fluorescent P-glycoprotein substrate rhodamine 123 (R123) (AnaSpec). 20 μL of 20 μM R123 diluted in 1x Dulbecco’s PBS was added to each well and the plate was incubated at 37°C with 5% CO_2_ for 1 hr. Fluorescence from each well was measured in a spectrophotometer at excitation wavelength of 488 nm and emission wavelength of 530 nm.

### Hoechst 33342 assay

To further assess the efflux function of P-gp transporters in microfilariae, the fluorescent P-glycoprotein substrate Hoechst 33342 was co-incubated with the macrocyclic lactones and microfilariae. Dilutions of macrocyclic lactones were incubated with 300 microfilariae per well in RPMI1640 at 37°C with 5% CO_2_ for 90 min. 20 μL of 20 μM Hoechst 33342 (ThermoScientific) in 1x Dulbecco’s PBS was added to each well and the plate was incubated for 10 min at room temperature. Fluorescence from each well was measured in a spectrophotometer at excitation wavelength of 361 nm and emission wavelength of 486 nm.

### Statistical analysis

One-way or two-way ANOVAs were performed to analyze absorbance, fluorescence and luminescence values obtained as assay outputs. A tukey test was used to compare the means of groups. All statistical analyses were performed on GraphPad Prism 8.

## Results

### Cell membrane integrity assays

#### Trypan blue staining is higher in Missouri isolate compared to resistant isolates

**Fig 1:**
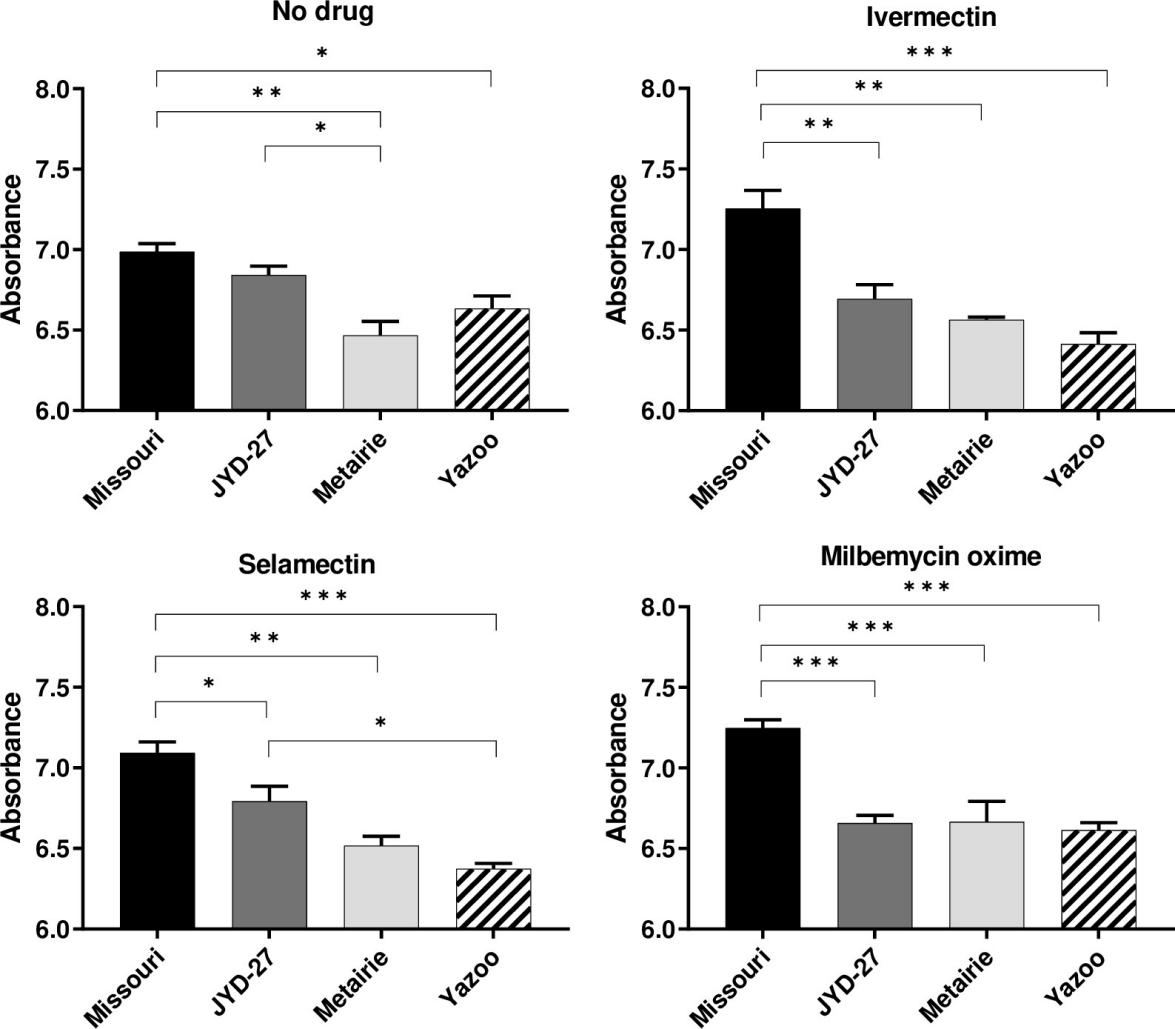
Trypan blue staining. Absorbance values (mean ± SE) obtained by incubating trypan blue and microfilariae after incubation with no drugs, 1μM of ivermectin, 1μM of selamectin and 1μM of milbemycin oxime for 1 hr at 37°C. (Asterisks indicate differences between strains at a given drug dose, * p < 0.05, ** p < 0.01, *** p < 0.001).

Trypan blue is commonly used to detect and selectively stain dead cells that have damaged membranes [16]. The polysulphated hydrocarbon dye- trypan blue enters a cell upon membrane damage and has previously been used to stain anatomic components of nematodes [16,19]. We found a linear relationship between absorbance and number of stained microfilariae without removing excess dye (S1 Fig); this indicated measurement of intracellular proteins complexed with trypan blue. In the presence of MLs, the susceptible Missouri isolate had higher absorbance values when compared to ML-resistant isolates JYD-27, Metairie and Yazoo (p < 0.05, Fig 1). A similar trend was observed in untreated microfilariae, although the difference between drug susceptible and resistant isolates was not as large as that observed in the presence of MLs. While trypan blue staining had a dose-dependent relationship with ML treatment, the apparent EC_50_ was similar for all isolates tested (S2 Fig).

#### Propidium iodide staining is elevated in Yazoo isolate in the presence of MLs

**Fig 2:**
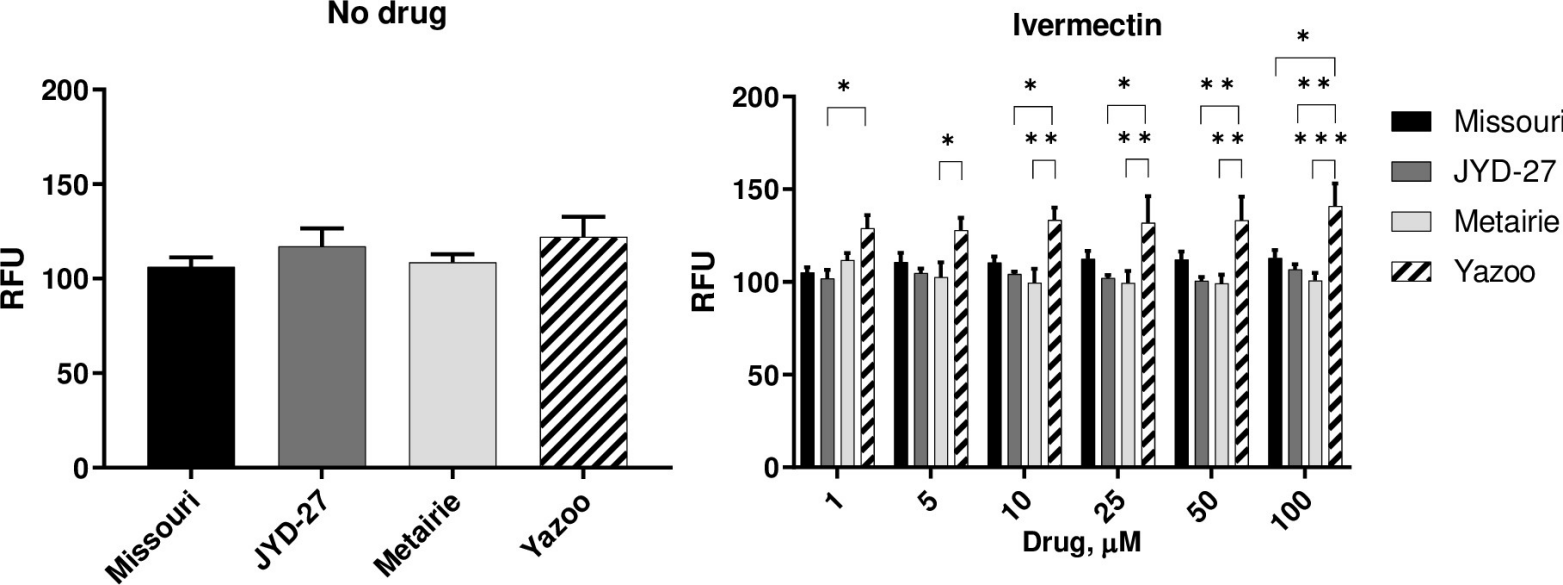
Propidium iodide staining. Fluorescence values (mean ± SE) obtained by incubating propidium iodide and microfilariae after pre-incubation with no drugs or ivermectin for 1 hr at 37°C. (Asterisks indicate differences between strains at a given drug dose, * p < 0.05, ** p < 0.01, *** p < 0.001).

Propidium iodide is another stain that is used to identify cells that have permeable membranes [16]. Propidium iodide is a nucleic acid intercalating agent that is also excluded from viable cells. Following intercalation, the excitation/emission maxima change, allowing for the specific measurement of intracellular staining. In some studies, *C*. *elegans* viability has been assessed using this method [20,21]. In our assays, there were no significant differences among *D*. *immitis* isolates for fluorescence values when live microfilariae were incubated with propidium iodide in the absence of MLs (Fig 2). Under a range of ivermectin concentrations, the resistant isolate Yazoo had significantly increased fluorescence values compared to the other isolates, with the most pronounced difference observed at 100μM (p < 0.05, Fig 2).

There were no significant differences in pairwise comparisons of fluorescence when selamectin was used except that Yazoo was higher than Metairie at 5μM concentration (p = 0.016). For milbemycin oxime, there were no significant differences in pairwise comparisons except Yazoo was higher than Metairie at 10μM concentration (p = 0.0329). Typical sigmoid dose-response curves were not observed over the range of drug concentrations tested (Fig 2, S3 Fig).

### Metabolism assays

#### Missouri isolate has higher metabolic activity as measured by resazurin metabolism

**Fig 3:**
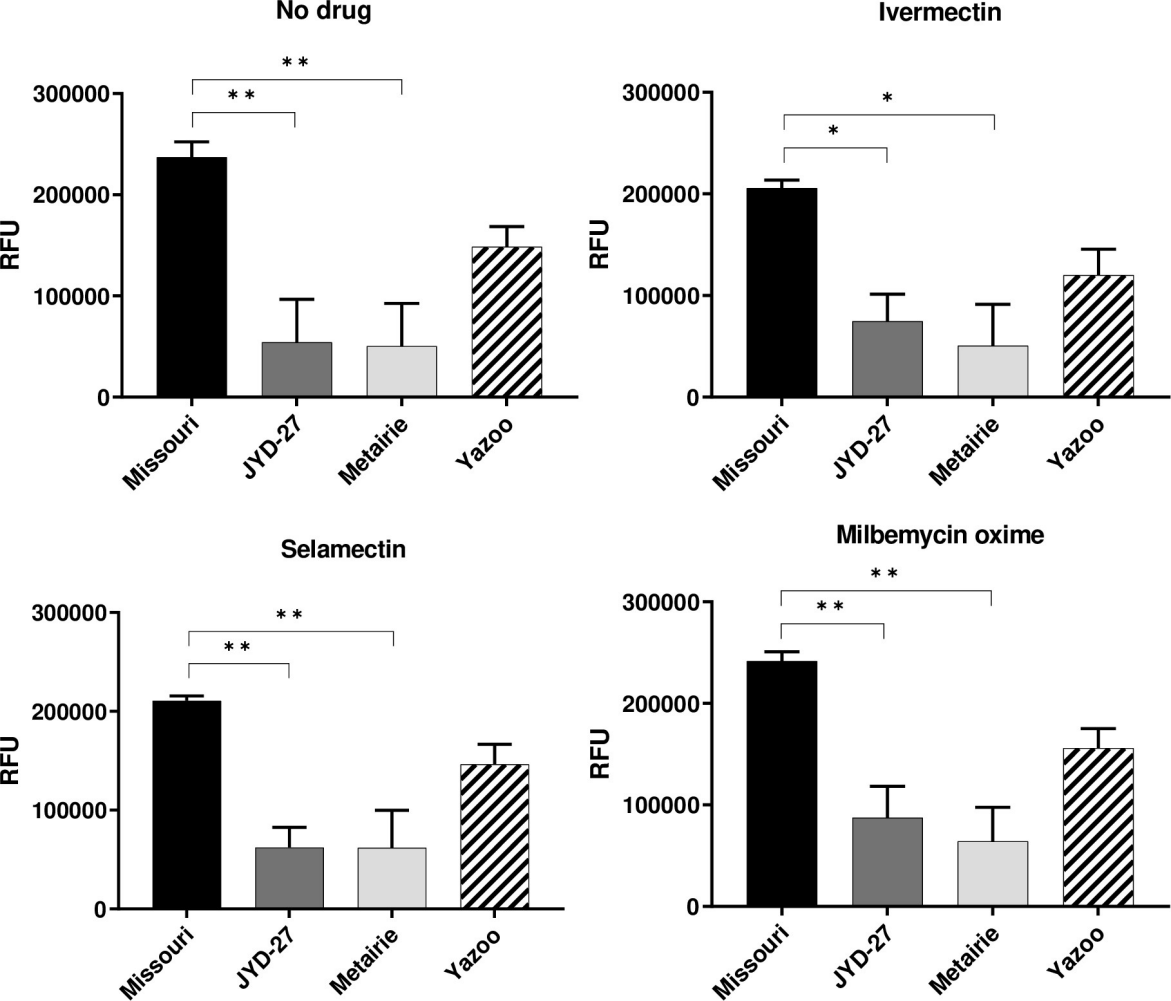
Resazurin metabolism. Fluorescence values (mean ± SE) obtained by incubating resazurin and microfilariae after incubation with no drugs, 1μM of ivermectin, 1μM of selamectin and 1μM of milbemycin oxime for 1 hr at 37°C. (Asterisks indicate differences between strains at a given drug dose, * p < 0.05, ** p < 0.01).

The metabolism assays used in this study, Resazurin (Alamar blue) and MTS, are dyes that allow the measurement of cellular redox activity. Non-fluorescent resazurin is reduced to fluorescent resorufin as early as 1 hour in helminth studies [22]. Investigators have used the reduction of resazurin to resorufin as a measure of metabolism for cell viability assays and as an alternative to motility [23]. In the present study, the different isolates exhibited large variation in resazurin metabolism in the absence of MLs, with the drug- susceptible Missouri isolate having significantly higher values when compared to the resistant isolates JYD-27 or Metairie (Fig 3). The pattern of resorufin metabolism was consistent in the presence of 1μM MLs, with Missouri having significantly higher values than JYD-27 and Metairie (Fig 3). Yazoo microfilariae reduced resazurin less than Missouri microfilariae, but more than JYD-27 and Metairie microfilariae (Fig 3). However, these differences were not statistically significant. A similar pattern of resazurin metabolism was observed in the presence of MLs. Concentration of MLs did not have a dose-dependent relationship with resazurin metabolism (S4 Fig).

#### MTS Tetrazolium assay is not suitable for distinguishing differences among strains

MTS tetrazolium in the presence of phenazine methosulfate is a modified MTT assay, in which the membrane-permeable tetrazolium salt is reduced to a dark water-soluble formazan product by metabolically active cells which can be measured spectrophotometrically [24]. MTT has long been used in *in vitro* nematode viability assays [24,25]. Co-incubation with MLs caused absorbance values to increase in a dose-dependent manner (S5 Fig), but no differences were observed among isolates incubated with the MTS reagent in the absence of drugs (S5 Fig). Following this result, no further MTS assays were pursued.

#### Luciferase assay detects low levels of ATP after bead beating

**Fig 4:**
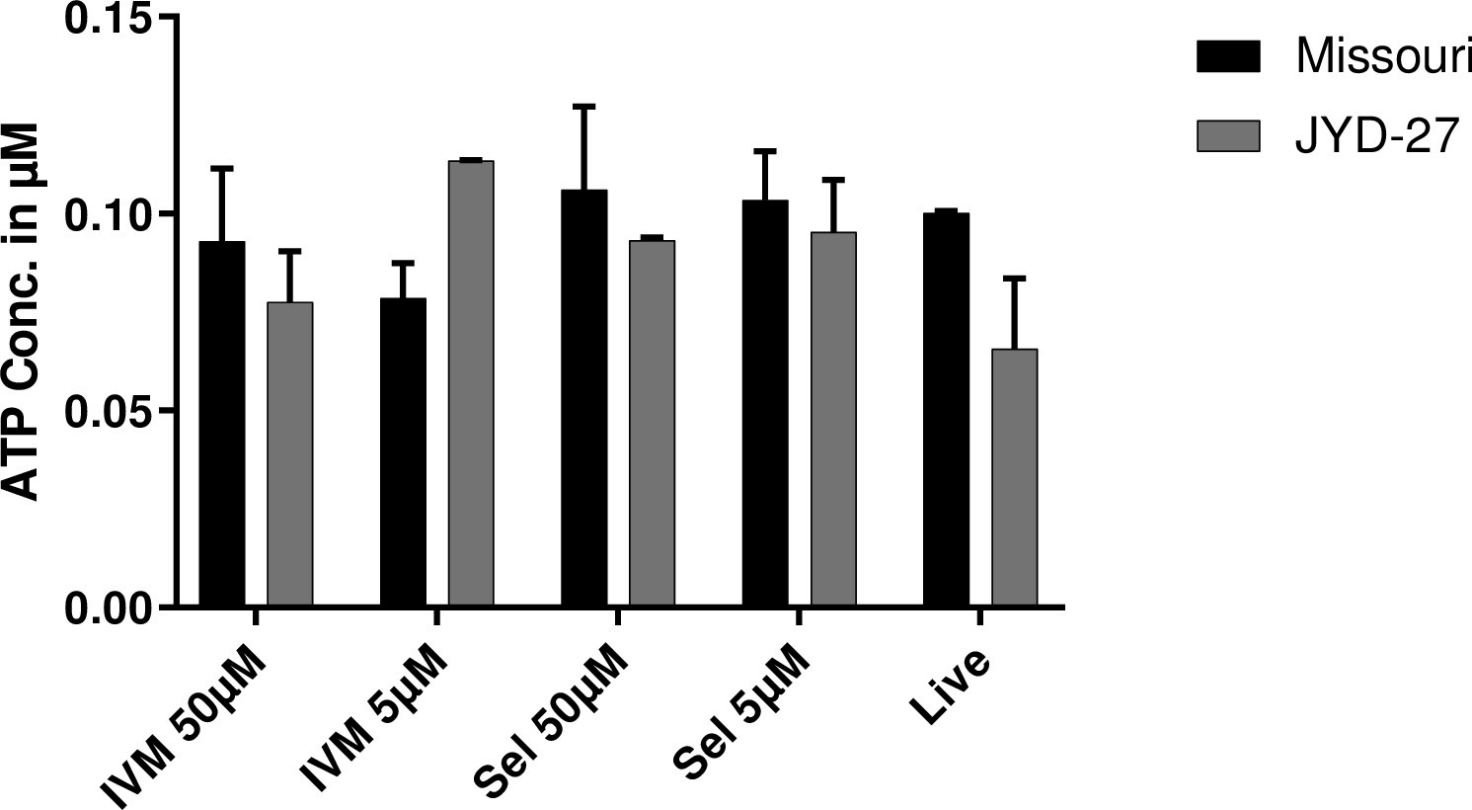
Recombinant luciferase assay. ATP concentrations (mean ± SE) obtained by interpolating from the standard curve. Luminescence values used to calculate ATP concentrations were obtained by incubating luciferase with microfilariae with dilutions of macrocyclic lactones drugs for 1 hr at 37°C.

Bioluminescence based quantitative measurements of ATP have been used as indicators of nematode metabolism and survival [26]. The amount of luminescence is directly proportional to the number of viable cells present prior to reagent-induced cell lysis. Attempted uses of ATP/luciferase assays to assess drug induced death in helminths suffer from the inability of the reagent to induce lysis [27]. Initial investigation revealed that the ATP reagent used was unable to cause lysis of microfilariae. However, bead beating was adequate to release cytoplasmic contents and the supernatant could be used in the luciferase assay. A standard curve of luminescence values was obtained with serial dilutions of ATP (R^2^ = 0.999) (S6 Fig). No significant differences in luminescence were observed between JYD-27 and Missouri isolates incubated with or without MLs (Fig 4).

### P-glycoprotein assays

#### R123 efflux activity is increased in Metairie and Yazoo isolates

**Fig 5:**
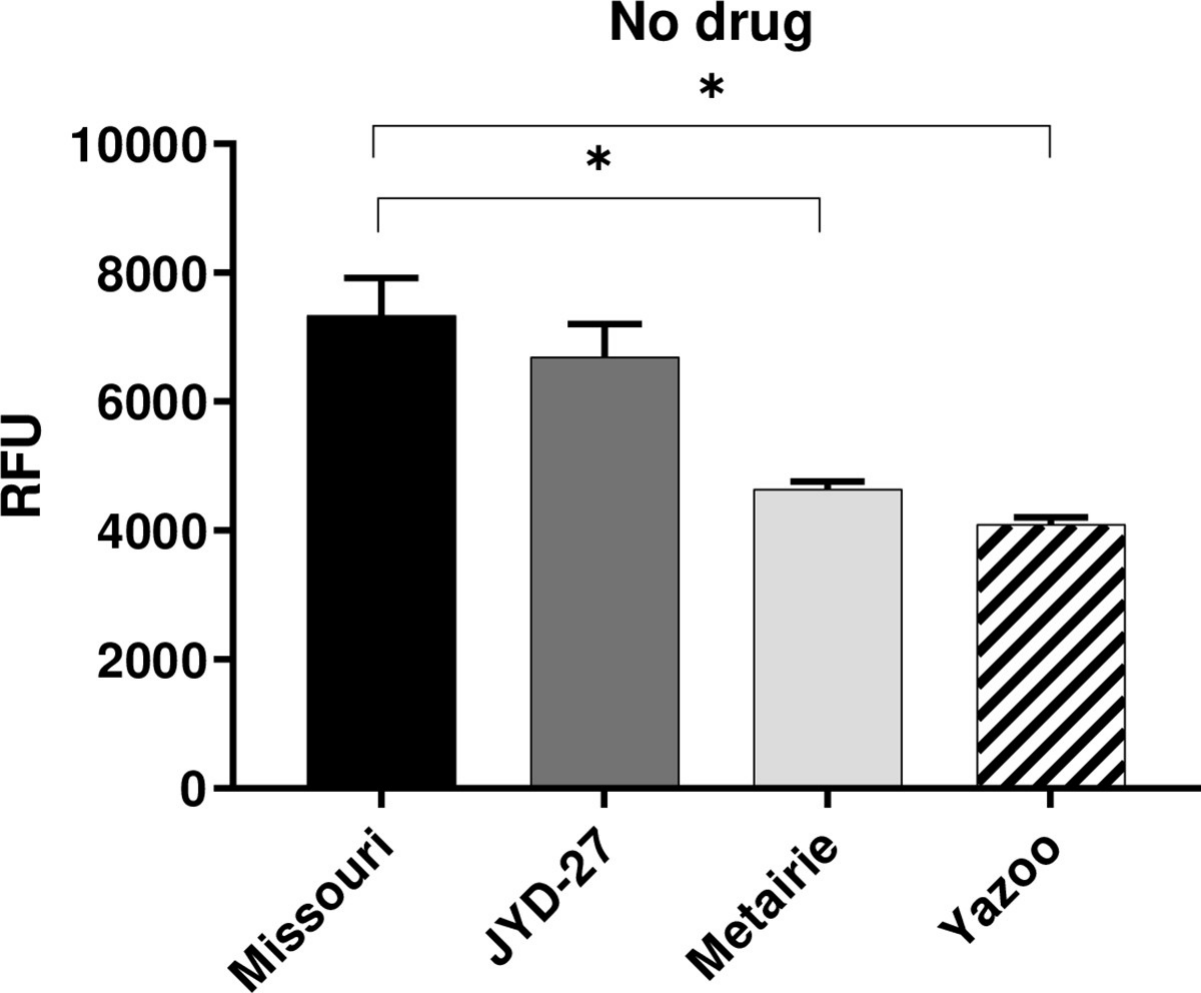
R123 efflux assay. Fluorescence values (mean ± SE) obtained by incubating Rhodamine 123 and microfilariae in media in the absence of drugs for 1 hr at 37°C. (Asterisks indicate differences between strains p < 0.05).

Rhodamine 123 is a fluorescent substrate that binds to the R site on P-gp and is effluxed by mammalian and nematode P-gps [28]. In the absence of drugs, Metairie and Yazoo had increased P-gp activity indicated by lower levels of intracellular R123 compared to the Missouri isolate (P<0.05, Fig 5). However, P-gp mediated efflux activity of JYD-27 was indistinguishable from Missouri. Efflux assays conducted in the presence of MLs revealed no differences among isolates, and fluorescence values did not have a dose-response relationship with the MLs (S7 Fig).

#### H33342 efflux activity is decreased in Yazoo isolate

**Fig 6:**
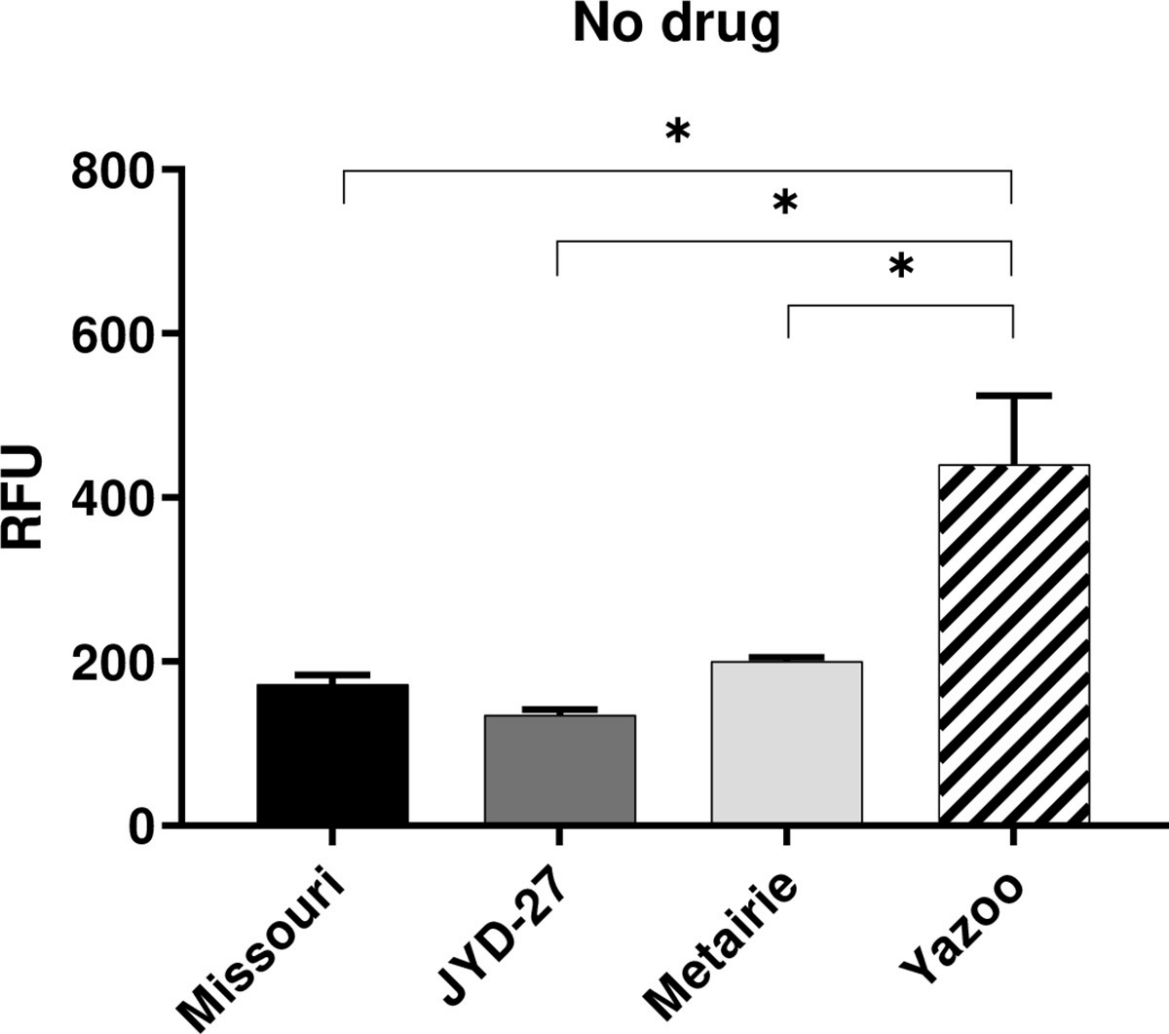
H3342 efflux assay. Fluorescence values (mean ± SE) obtained by incubating Hoechst 33342 and microfilariae in media in the absence of drugs for 1 hr at 37°C. (Asterisks indicate differences between strains p < 0.05).

Hoechst 33342 is a fluorescent substrate that binds to the H site on P-gp and is effluxed by mammalian and nematode P-gp [28]. Constitutive P-gp-mediated H33342 efflux activity was higher in Missouri, JYD-27, and Metairie, with reduced P-gp activity in Yazoo (Fig 6). Co-incubation with MLs reduced the amount of P-gp activity in a dose-dependent manner, with P-gp activity that was lower in the Missouri isolate at high doses of MLs (S8 Fig). Dose-response experiments were not performed with Yazoo and Metairie for H33342 due to availability of sufficient microfilariae during the study period.

## Discussion

The incidence of dirofilariosis is increasing in the United States [29,30]. Concurrently, there is a rise in the number of lack of efficacy (LOE) reports made by veterinarians [9]. The ability to distinguish compliance failure and true ML resistance is a necessity, and using experimental infections to determine ML susceptibility status is both difficult and undesirable. An *in vitro* assay using microfilariae as a surrogate to measure ML susceptibility could provide useful prognostic information to clinicians and harmonize basic drug discovery studies.

Our experiments revealed that several assays could detect isolate-specific differences following exposure to MLs, but that these differences were not always predictive of clinical susceptibility status. For example, the susceptible isolate (Missouri) had increased trypan blue staining as compared to the resistant isolates (JYD-27, Metairie, Yazoo) following exposure to 1μM macrocyclic lactones. However, this trend was also present in the absence of MLs (Fig 1). Unexpectedly, propidium iodide staining was increased in Yazoo compared to other isolates in the presence of MLs. Significant differences in resazurin reduction in the absence of drugs suggest that basal metabolic levels vary among isolates. The susceptible Missouri isolate had higher basal metabolism and was able to reduce resazurin at higher levels than the resistant isolates JYD-27 and Metairie, but not Yazoo. These patterns were similar in the presence of MLs and were surprising since we expected the resistant isolates to have increased metabolism in the presence of MLs. Taken together our data underscores the need to test a broad range of isolates when investigating methods for detecting resistance. Clearly, some isolate-specific differences can be detected these methods are not capable of detecting all resistant isolates under the conditions tested. However, it is quite possible that these assays could be further optimized with different temperatures or drug exposure times, resulting in more definitive outcomes.

P-gps contribute to drug resistant phenotypes in several nematode parasites and have been implicated in *D*. *immitis* resistance to MLs [18,31,32]. P-gps are transmembrane pumps that efflux xenobiotics including MLs across membranes and are expressed in both the nematode intestine and body wall [33,34]. R123 and H33342 are fluorescent substrates of P-gp and accumulate in the mitochondria or nucleus, respectively, if they are not removed via P-gp mediated efflux activity [35]. The two dyes bind to different sites on P-gp molecules [28,36]. Using R123, we found that basal levels of P-gp efflux activity were higher in Metairie and Yazoo as compared to Missouri. On the other hand, in the H33342 assays, there was increased P-gp activity in Missouri, JYD-27, and Metairie. The different results with the two P-gp substrates may indicate different repertoires of P-gp gene expression and substrate specificity, since there are numerous P-gp genes expressed by *D*. *immitis* and the two substrates tested bind different sites [37]. We hypothesize this finding indicates different profiles of P-gp expression or gene polymorphisms at R123 and/or H33342 binding sites [38,39]. Our findings were consistent with previous studies of *Haemonchus contortus* where P-gp is up-regulated in some but not all resistant isolates [40,41]. Whole organism P-gp assays are useful for understanding the net P-gp activity in an organism, but the differences among isolates and assays suggests that individual P-gps should also be studied in isolation to further understand pharmacological differences.

This study investigated membrane permeability, metabolic activity, and P-gp activity to measure ML-mediated killing of microfilariae among different *D*. *immitis* isolates. In some cases, assays could detect differences among resistant and susceptible isolates, but no single test clearly discriminated all resistant and susceptible isolates. Interestingly, there were also differences among the resistant isolates tested. These diverse phenotypes reinforce the importance of evaluating multiple isolates during drug screening if microfilariae are used. Our results suggest that further optimization is needed before any of the tested assays could be used to detect resistance in a clinical setting. In our studies, we only evaluated worms following 1 hr of drug exposure for clinical convenience. It is likely that longer co-incubation times could lead to more definitive phenotypic differences among isolates. Our results suggest there is a need to explore the assays under a variety of conditions such as time or temperature.

Altogether, our studies suggest that phenotypic differences exist among isolates of *D*. *immitis*, and this may confound the laboratory detection of resistance using methods developed for detecting death in cell cultures. Importantly, investigators should be cautious when using such assays in anti-parasitic drug screening, as they may not reveal the true “live” or “dead” status of the parasite. Assessment of biochemical phenotypes of regional *D*. *immitis* populations under refined conditions will be helpful for improvement of these assays. Such studies may also provide phenotypic clues leading to future hypothesis-driven discovery of ML drug resistance mechanisms.

## Supporting information

S1 Fig Trypan blue optimization. Absorbance values of different numbers of microfilaria incubated with 0.04% trypan blue solution. Linear fit, R^2^ = 0.93.
(DOCX)

S2 Fig Trypan blue staining under different drug concentrations. Absorbance values (mean ± SE) obtained by incubating trypan blue and microfilariae after incubation with different dilutions of ivermectin, selamectin and milbemycin oxime for 1 hr at 37°C.
(DOCX)

S3 Fig Propidium iodide staining. Fluorescence values (mean ± SE) obtained by incubating propidium iodide and microfilariae after incubation with different dilutions of selamectin and milbemycin oxime for 1 hr at 37°C. (* indicates a difference between isolates at a given drug dose, p < 0.05).
(DOCX)

S4 Fig Resazurin metabolism. Fluorescence values (mean ± SE) obtained by incubating resazurin and microfilariae after incubation with different dilutions of ivermectin, selamectin and milbemycin oxime for 1 hour at 37°C. All values significantly different (p < 0.05) except Selamectin at 100μM.
(DOCX)

S5 Fig MTS Assay. Absorbance values (mean ± SE) obtained by incubating MTS and microfilariae after incubation with different dilutions of ivermectin, selamectin and milbemycin oxime for 1 hour at 37°C.
(DOCX)

S6 Fig Recombinant luciferase optimization. Luminescence values obtained with a standard curve of pure ATP. Non-linear fit, R^2^ = 0.9999.
(DOCX)

S7 Fig Fluorescence values (mean ± SE) obtained by incubating Rhodamine123 and microfilariae after incubation with different dilutions of ivermectin, selamectin and milbemycin oxime for 1 hour at 37°C. (DOCX)

S8 Fig Fluorescence values (mean ± SE) obtained by incubating Hoechst33342 and microfilariae after incubation with different dilutions of ivermectin, selamectin and milbemycin oxime for 1 hour at 37°C. (Asterisks indicate differences between strains at a given drug dose, * p < 0.05, ** p < 0.01, *** p < 0.001, **** p < 0.0001).
(DOCX)
