# Supplementary material for: Laboratory assays reveal diverse phenotypes among microfilariae of Dirofilaria immitis isolates with known macrocyclic lactone susceptibility status
Source: PLoS One. 2020 Aug 6;15(8):e0237150. doi: 10.1371/journal.pone.0237150 (PMC7410292; doi:10.1371/journal.pone.0237150)
Supplement: S1 Fig — Absorbance values of different numbers of microfilaria incubated with 0.04% trypan blue solution. Linear fit, R2 = 0.93. (DOCX) [file pone.0237150.s001.docx]

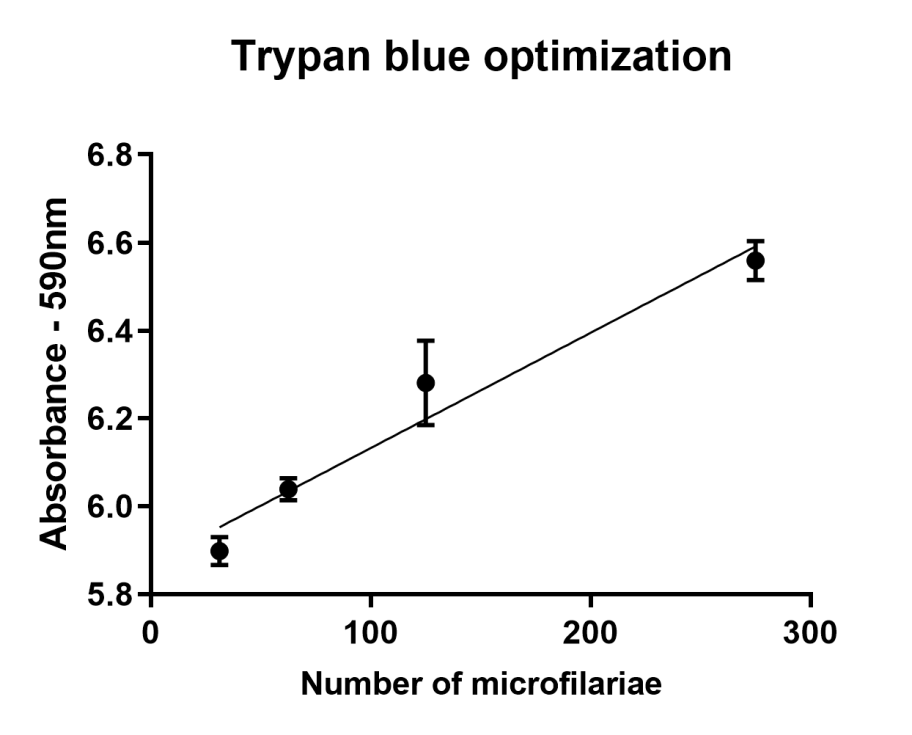


S1 Fig. Trypan blue optimization. Absorbance values of different numbers of microfilaria incubated with 0.04% trypan blue solution. Linear fit, R^2^= 0.93
