# Supplementary material for: Laboratory assays reveal diverse phenotypes among microfilariae of Dirofilaria immitis isolates with known macrocyclic lactone susceptibility status
Source: PLoS One. 2020 Aug 6;15(8):e0237150. doi: 10.1371/journal.pone.0237150 (PMC7410292; doi:10.1371/journal.pone.0237150)
Supplement: S2 Fig — Absorbance values (mean ± SE) obtained by incubating trypan blue and microfilariae after incubation with different dilutions of ivermectin, selamectin and milbemycin oxime for 1 hr at 37°C. (DOCX) [file pone.0237150.s002.docx]

S2 Fig. Trypan blue staining under different drug concentrations. Absorbance values (mean ± SE) obtained by incubating trypan blue and microfilariae after incubation with different dilutions of ivermectin, selamectin and milbemycin oxime for 1 hr at 37°C.
