# Supplementary material for: Laboratory assays reveal diverse phenotypes among microfilariae of Dirofilaria immitis isolates with known macrocyclic lactone susceptibility status
Source: PLoS One. 2020 Aug 6;15(8):e0237150. doi: 10.1371/journal.pone.0237150 (PMC7410292; doi:10.1371/journal.pone.0237150)
Supplement: S3 Fig — Fluorescence values (mean ± SE) obtained by incubating propidium iodide and microfilariae after incubation with different dilutions of selamectin and milbemycin oxime for 1 hr at 37°C. (* indicates a difference between isolates at a given drug dose, p < 0.05). (DOCX) [file pone.0237150.s003.docx]

S3 Fig. Propidium iodide staining. Fluorescence values (mean ± SE) obtained by incubating propidium iodide and microfilariae after incubation with different dilutions of selamectin and milbemycin oxime for 1 hr at 37°C. (* indicates a difference between isolates at a given drug dose, p < 0.05).
