# Supplementary material for: Laboratory assays reveal diverse phenotypes among microfilariae of Dirofilaria immitis isolates with known macrocyclic lactone susceptibility status
Source: PLoS One. 2020 Aug 6;15(8):e0237150. doi: 10.1371/journal.pone.0237150 (PMC7410292; doi:10.1371/journal.pone.0237150)
Supplement: S4 Fig — Fluorescence values (mean ± SE) obtained by incubating resazurin and microfilariae after incubation with different dilutions of ivermectin, selamectin and milbemycin oxime for 1 hour at 37°C. All values significantly different (p < 0.05) except Selamectin at 100μM. (DOCX) [file pone.0237150.s004.docx]

S4 Fig. Resazurin metabolism. Fluorescence values (mean ± SE) obtained by incubating resazurin and microfilariae after incubation with different dilutions of ivermectin, selamectin and milbemycin oxime for 1 hour at 37°C. All values significantly different (p < 0.05) except Selamectin at 100µM.
