# Supplementary material for: Laboratory assays reveal diverse phenotypes among microfilariae of Dirofilaria immitis isolates with known macrocyclic lactone susceptibility status
Source: PLoS One. 2020 Aug 6;15(8):e0237150. doi: 10.1371/journal.pone.0237150 (PMC7410292; doi:10.1371/journal.pone.0237150)
Supplement: S6 Fig — Luminescence values obtained with a standard curve of pure ATP. Non-linear fit, R2 = 0.9999. (DOCX) [file pone.0237150.s006.docx]

S6 Fig. Recombinant luciferase optimization. Luminescence values obtained with a standard curve of pure ATP. Non-linear fit, R^2^ = 0.9999.
