# Supplementary material for: Laboratory assays reveal diverse phenotypes among microfilariae of Dirofilaria immitis isolates with known macrocyclic lactone susceptibility status
Source: PLoS One. 2020 Aug 6;15(8):e0237150. doi: 10.1371/journal.pone.0237150 (PMC7410292; doi:10.1371/journal.pone.0237150)
Supplement: S8 Fig — (Asterisks indicate differences between strains at a given drug dose, * p < 0.05, ** p < 0.01, *** p < 0.001, **** p < 0.0001). (DOCX) [file pone.0237150.s008.docx]

S8 Fig. Fluorescence values (mean ± SE) obtained by incubating Hoechst33342 and microfilariae after incubation with different dilutions of ivermectin, selamectin and milbemycin oxime for 1 hour at 37°C. (Asterisks indicate differences between strains at a given drug dose, * p < 0.05, ** p < 0.01, *** p < 0.001, **** p < 0.0001).
